# Boys don’t try? Gendered stigma specifically reduces help-seeking for disordered eating in men, but not women

**DOI:** 10.1186/s40337-025-01407-7

**Published:** 2025-09-17

**Authors:** Martin S. Lehe, Georg Halbeisen, Vanessa C. Juergensen, Luisa Sabel, Sabine Steins-Loeber, Georgios Paslakis

**Affiliations:** 1 Medical Faculty, University Clinic for Psychosomatic Medicine and Psychotherapy, Ruhr-University Bochum, Campus East-Westphalia, Virchowstr. 65, 32312 Lübbecke, Germany; 2 Department of Clinical Psychology and Psychotherapy, Otto-Friedrich- University of Bamberg, Markusplatz 3, 96047 Bamberg, Germany

**Keywords:** Eating disorders, Men, Masculinity, Stigma, Gender, Treatment barrier, Access to care, Help-Seeking

## Abstract

**Background:**

Eating disorders (EDs) affect individuals across all genders, but men remain underrepresented in ED treatment settings. Stigma related to EDs in men may impede help-seeking, particularly for symptoms that deviate from traditional masculine ideals. This study investigates whether stigma-related perceptions of EDs in men specifically moderate the association between disordered eating symptoms and help-seeking intentions in men, i.e., whether there is a gender-specific component of ED stigma.

**Methods:**

In a cross-sectional survey, *n* = 242 men and *n* = 249 women completed questionnaires on various disordered eating symptoms (“traditional” thinness-oriented, muscularity-oriented, orthorexic, and avoidant/restrictive eating behaviors), stigma-related perceptions of EDs in men, and help-seeking intentions. Moderator analyses were conducted by gender to explore interactions between symptom severity and stigma in predicting help-seeking intentions.

**Results:**

Help-seeking intentions increased with the severity of disordered eating symptoms in both men and women, except for avoidant/restrictive eating behavior in both genders and muscularity-oriented symptoms in men. Stigma-related perceptions of EDs in men moderated the association between symptoms and help-seeking intentions in men, which was exclusively the case for “feminized” ED symptoms (i.e., thinness and weight concerns). No moderation effects were observed for other symptom domains or among women.

**Conclusions:**

Results support the notion of a gender-specific role of stigma in men’s help-seeking behaviors for EDs. Such stigma, which seems to be related to “feminized” ED symptoms, may contribute to men’s reluctance to seek help for EDs. These findings emphasize the need for interventions tailored to reduce stigma, particularly regarding men’s experiences of EDs, to support equal access to healthcare.

**Supplementary Information:**

The online version contains supplementary material available at 10.1186/s40337-025-01407-7.

## Abstract (plain language summary)

Eating disorders are generally considered to be a “women's disease”, but statistics show that more men are affected. Women, and to a greater extent men, are less likely to seek help for an eating disorder from healthcare services. Stigma seems to be a barrier, according to previous research. In this study, we investigated whether there is a potential gender-specific effect of stigma of eating disorders (i.e. among others EDs as “women's disorders”) on men’s help-seeking. We found that stigma was associated with reduced help-seeking among men for eating disorder-related symptoms consistent with a stereotypical 'feminized' construction of eating disorders, but not among women. Stigma thus appears to be associated with reduced use of healthcare services for eating disorders, whereby men may face gender-specific challenges. This calls for further efforts to reduce eating disorder stigma.

**Supplementary Information:**

The online version contains supplementary material available at 10.1186/s40337-025-01407-7.

## Background

Eating disorders (EDs) are severe psychiatric conditions characterized by altered eating behaviors, distorted body image perceptions, and dysfunctional weight-control behaviors [1]. They are associated with significant physical and psychological impairments and higher mortality rates compared to other mental disorders [2]. Deeply rooted in historical and cultural contexts with patriarchal standards of beauty [3, 4], EDs have long been perceived as predominantly affecting women, despite seminal case reports in men [5–7]. This perspective also influenced the diagnostic criteria and instruments, which have primarily been developed in women’s samples [8], further limiting the early identification of and intervention for EDs in men [9, 10]. However, there is nowadays a consensus that EDs can affect individuals of all genders and social backgrounds, including adult men [8, 11, 12]. Epidemiological data from the last three decades indicate a 22% increase in EDs among men, compared to a 12% increase among women [13]. Although women remain the majority, current lifetime prevalence estimates of 2.2% in men compared to 8.4% in women suggest that men may account for one in four cases [14, 15].

However, men with EDs remain underrepresented in specialized treatment settings [16]. One contributing factor is lower help-seeking rates for EDs among men compared to women [17–20]. Men, especially when not meeting full diagnostic criteria [21] or presenting with lesser-known ED symptoms [e.g., exercise dependence, muscularity-related body image concerns, orthorexic and avoidant/restrictive eating symptoms [22–27], are less inclined to seek help compared to women. Timely help-seeking is important, since longer duration of an untreated ED may result in poorer long-term treatment and mental health outcomes [28], and illness chronicity is associated with higher mortality [29]. In order to ensure timely treatment, it is crucial to elucidate barriers to (men’s) help-seeking for EDs.

## Barriers to help-seeking for disordered eating in men

Help-seeking is a complex, multi-stepped process [30–32]. Individuals need to evaluate present symptoms, recognize and acknowledge their problem, gather information on help, weigh available alternatives against their costs, decide on seeking help, and finally translate this decision into action. Any disruption to this process could delay or prevent seeking help. Although men and women appear to recognize own ED symptoms equally [16], previous research points towards gender differences in other relevant domains, such as (a) knowledge and perception of EDs (lack of knowledge, reduced health literacy, fear of treatment, past negative experiences with treatment), (b) emotional barriers (shame, denial, emotional readiness), (c) practical barriers (cost of treatment, logistical challenges, waiting lists, lack of specialized services), and (d) social and structural barriers (lack of social support, societal norms, stigma and fear of judgment) [33–36]. It is also important to acknowledge that these barriers can be further differentiated within gender groups, for example, when considering the unique barriers of men and women with intersectional identities (e.g., sexual and/or ethnic minority men [37]), although this study focusses only on global gender differences.

Indeed, previous research found that men report stigma to specifically delay their help-seeking behavior for EDs [38, 39], raising the question of a possible gender-specific component of ED stigma. Stigma refers to the association of a certain characteristic of a person or group with a negative social stereotype or prejudice and is associated with a loss of social status for the stigmatized person, which may lead to discrimination [40]. Gender may be understood as a social construct, shaped by norms, roles, and expectations within a given culture or society. It refers to the socially constructed characteristics associated with being women, men, girls, and boys, including their behaviors, roles, and relationships [41]. For men, the internalized concepts of male gender and masculinity have been linked to delayed help-seeking for health-related issues [42–45]. An individual’s need or intention to seek help may conflict with internalized masculinity norms advocating dominance, emotional restraint, toughness, independence, and competitiveness [46], and bear the risk of social backlash [47]. Accordingly, both men and women were found at risk for healthcare avoidance due to problem minimization, doctor distrust, or privacy concerns driven by internalized masculinity norms [48] and showing ED stigmatization when conforming to masculine norms [49].

Seeking help may not only contradict traditional masculinity norms and gender roles but also threaten a man’s social status, given the perspective that manhood may be viewed as a precarious status that can be jeopardized by engaging in behaviors perceived as “feminine” or “insufficiently masculine” [50, 51]. These facets, along with the fear of losing social status for violating gender norms, may contribute to a perceived stigma surrounding EDs in men. This stigma could discourage men from seeking help, particularly when EDs are perceived as ”women’s conditions”.

In order to examine the link between stigma and men’s help-seeking for EDs, we recently explored whether stigma‑related perceptions of EDs in men (e.g., “It is better for a man to not admit having an eating disorder”) are associated with reduced help‑seeking intentions for a broad range of disordered eating symptoms [52]. We found that higher perceived stigma reduced men’s help-seeking intentions for traditionally “feminized” ED symptoms (i.e., thinness-oriented disordered eating) but not for muscularity-oriented, orthorexic, or avoidant/restrictive disordered eating behaviors, which seem to appear less gendered [53–55]. Notwithstanding the influence of other known factors such as delayed symptom recognition by health care professionals and/or the affected men themselves (see above), these findings could suggest that men might also lower their help-seeking intentions specifically due to expected stigmatization based on their gender. However, as our study focused exclusively on men, this interpretation remains preliminary. Men, for example, could have been discouraged from seeking help simply because they perceived EDs to be stigmatized in general or health care services to be unavailable. In other words, the assessment in our previous study may have simply captured a more general negative attitude towards people with EDs or mental health care services. In this case, this would be expected to affect help-seeking intentions across individuals of both genders. Thus, to argue for the gender-specificity of the effect of the proposed stigma facet, it is crucial to compare the effect of stigma toward men’s EDs between a sample of men and those individuals unlikely to be affected by men-oriented stigmatization, i.e., women. If our assessment specifically captures perceived stigma towards men’s EDs, rather than a general negative evaluation of having an ED or accessing mental health care services, we would expect a specific impact on men’s help-seeking intentions and that these attitudes would not predict help-seeking in women. Based on these considerations, we hypothesized that perceived stigma toward men’s EDs reduces help-seeking intentions in men but not in women.

## The present study

Based on the assumed positive association between ED symptoms and intentions to seek help in both genders as well as the proposed potentially men-specific facet of ED stigma, the present study investigated the hypothesis that perceived stigma of EDs in men reduces the effect of ED symptoms on intentions to seek help in men but not in women. This would point towards a gender-specific facet of ED stigma and contribute to our understanding of men’s reduced help-seeking behaviors for EDs and could inform more tailored intervention strategies. Given the differences in ED symptom presentation between men and women, we explored the effects of a broad range of ED-related symptoms on help-seeking intentions. Replicating the procedure of Lehe et al. [52], we assessed ED symptoms, intentions to seek help, and perceived stigma of EDs in men in both men and women samples. Given its “gold standard” character, i.e., wide application and broad validation in various populations, we assessed “traditional” ED symptoms with the Eating Disorder Examination-Questionnaire (EDE-Q), in addition to orthorexic (Duesseldorf Orthorexia Scale; DOS) and muscularity-oriented (Muscle Dysmorphic Disorder Inventory; MDDI) symptoms, and avoidant/restrictive eating behavior (Eating Disorders in Youth-Questionnaire; EDY-Q) .

## Methods

### Participants and design

The present study is a pre-registered secondary data analysis of combined online surveys. We included *N* = 242 adult men (*M*_age_ = 32.8, *SD*_age_ = 11.1, age range: 18–73 years), from Lehe et al. [52] (*n* = 116) and Eschrich et al. [56] (*n* = 126), and *N* = 249 adult women (*M*_age_ = 28.7, *SD*_age_ = 9.6, age range: 19–64 years) from Jürgensen et al. [57]. See Table 2 for additional demographic information. Participants were recruited between May 2022 and June 2024 through advertisements on university campuses and in online social networks (48% of men, 36% of women), and through Prolific (www.prolific.com; 52% of men, 64% of women). Participants recruited via Prolific were compensated £4.50 for their participation; eligible university students received course credit. Most participants identified as heterosexual and indicated that they had no migration background, were single, and had received more than 12 years of formal education (see Table 2 for details).We only included cis-gender men or women [since gender-diverse populations are commonly found to be affected by multiple intersectional stigma and distinct stressors [58, 59], who answered “no” to ever having received a diagnosis for an ED, and with a BMI ≤ 35 kg/m², given that we were interested in predicting help-seeking intentions unaltered by prior ED-related treatment experiences or severe weight stigma [60]. The latter is more likely in individuals with strongly pronounced obesity, according to a study on weight stigma in men reporting that those who experienced weight stigma had a higher current BMI (29.30 vs. 26.49 kg/m²) and a higher highest-ever BMI (33.21 vs. 28.66 kg/m²) compared to their peers who did not report experiences of weight stigma [61].

The combined samples had a power of 0.80 to detect a small-to-medium-sized interaction effect (ρ = 0.10) according to Cohen’s criteria [62] at α = 0.05, based on a power analysis with G*Power version 3.1.9.7 [63]. An effect of this magnitude was observed in previous research [52] and required a minimum sample size of *N* = 132 for moderation analyses with one covariate. The surveys were approved by the Ethics Committee of the Ruhr-University Bochum’s Medical Faculty at Campus East-Westphalia (men: AZ 2022 − 910, April 21st, 2022; women: AZ 2022 − 910_1, March 15th, 2024), prospectively registered (men: https://aspredicted.org/5T3_NH5, women: 10.17605/OSF.IO/EFN4V), and conducted in accordance with the Declaration of Helsinki. All participants gave informed consent. Data and materials can be obtained from the corresponding author upon request.

### Measures and procedure

The surveys were administered on our web server using jsPsych [64] and introduced with the aim of enhancing the detection of disordered eating in men and women, respectively. Upon accessing the study’s website, participants were presented with the study information and consent forms. We explained that the survey encompassed the assessment of disordered eating behaviors and associated symptoms and that participants could receive feedback regarding their risk for an ED. Participants were required to initially provide sociodemographic information, ED history and treatment, and previous study participation. Further details can be found in Lehe et al. [52] and Eschrich et al. [56]. Previous participation served as a quality control measure to exclude data from individuals who participated repeatedly. The survey among women, which was conducted after the men´s surveys, included an additional attention check question at a random position within the survey [“Please mark the word giraffe” among a series of response options; [65]. Subsequently, participants completed an ED risk assessment [EAT-8]; [66], followed by the assessment of further disordered eating symptoms, help-seeking intentions, and stigma-related perceptions of EDs in men, presented in randomized order. The overall duration to complete the survey ranged from 15 to 20 min.

#### Eating disorder symptoms

We used the validated German version of the Eating Disorder Examination-Questionnaire [EDE-Q]; [67, 68] to assess ED symptoms within the last 28 days. The 22 attitudinal items are rated on a 7-point Likert scale from 0 (*never*) to 6 (*every day*) and are accompanied by six open-ended questions, with the latter not used in scale score computation. Given that previous studies did not support the proposed factor structure of the EDE-Q in men [69], we only report the overall mean score based on the attitudinal items (ω_men_ = 0.93; ω_women_ = 0.96).

Extending upon previous studies, we also assessed additional disordered eating and associated symptoms. First, the Duesseldorf Orthorexia Scale [DOS]; [70] was used to assess the preoccupation with healthy eating and related behaviors over the past week. The scale consists of ten items, each of which contains a statement (e.g., “I prioritize healthy eating over pleasure”) rated on a 4-point scale ranging from 1 (*this does not apply to me*) to 4 (*this applies to me*). The total score is computed as the sum of all items (ω_men_ = 0.85; ω_women_ = 0.86).

Second, symptoms related to avoidant/restrictive food intake disorder (ARFID) were assessed with the modified version of the Eating Disorders in Youth-Questionnaire [EDY-Q]; [71], which, despite the word “youth” in its name, has been adapted and validated for use in German-speaking adults [72]. The questionnaire consists of 14 items covering the three subscales food avoidance, selective eating, functional dysphagia, and issues related to underweight. Additional questions assessing pica, rumination, and weight/shape concerns (ARFID exclusion criteria) are not routinely used for scale score computation. Responses are recorded on a 7-point scale from 0 (*never true*) to 6 (*always true*). The total score was calculated as the mean of items 1 to 5 and 8 to 12 (ω_men_ = 0.66; ω_women_ = 0.55), while subscale scores were computed as the means of their respective items, following the established scoring conventions of the instrument.

Finally, the validated German version of the Muscle Dysmorphic Disorder Inventory [MDDI]; [73] was included, which assesses disturbances and behaviors associated with muscularity-related body image (e.g., the belief that one’s body is too skinny, hating one’s body, and experiencing depressed mood when not engaging in exercise). The instrument consists of 13 items rated on a 5-point scale from 1 (*never*) to 5 (*always*). The total score (ω_men_ = 0.82; ω_women_ = 0.70) and the scores for the three subscales (drive for size, appearance intolerance, and functional impairment; for reliability measures of subscales see Supplementary Material/Table S1) were computed as sum scores in accordance with the instrument’s convention.

#### Help-seeking intentions

Participants’ intentions to seek help were assessed using an item from the validated German version of the Stages of Change Questionnaire for Eating Disorders [SOCQ-ED]; [74]. The SOCQ-ED is grounded in the transtheoretical model of change [75]. It measures a participant’s motivation to change specific ED symptoms and the overall intention to seek treatment for disordered eating. Participants respond to each item on a 7-point scale that reflects the different motivational stages of the transtheoretical model of change (no need for therapy, precontemplation, contemplation, preparation, action, maintenance, termination; coded from 0 to 6).

#### Stigma-related perceptions of disordered eating in men

Due to the absence of an existing (German) measure on stigma-related perceptions of EDs in men, we previously developed a set of seven items based on the lived experiences of men with EDs, which demonstrated factorial validity [52, 76]. The items capture a diverse range of men’s perceived ED-related stigma (e.g., “Eating disorders are women’s diseases”, “Doctors take eating disorders less seriously in men than in women”, “It is better for a man to not admit having an eating disorder”). The items were rated on a 4-point scale from 1 (*completely disagree*) to 4 (*completely agree*) and aggregated as a total mean score (men: McDonald’s ω = 0.74; women: McDonald’s ω = 0.64), such that higher values indicated higher perceived stigma. A comprehensive list of the items is presented in Table 1.

**Table 1:** *Items and descriptive characteristics of the Self-Developed Stigma-Related perceptions of eating disorders in men measure*

Item	All				Men				Women				Wilcox
	M	SD	Min	Max	M	SD	Min	Max	M	SD	Min	Max	*p*
1. When men eat too little, are obese or exercise excessively, it is usually not due to an eating disorder.	2.23	0.75	1	4	2.43	0.72	1	4	2.04	0.72	1	4	< 0.001
2. Eating disorders are women’s diseases.	1.51	0.71	1	4	1.53	0.73	1	4	1.48	0.69	1	4	0.491
3. It is better for a man to not admit having an eating disorder.	1.27	0.62	1	4	1.43	0.76	1	4	1.12	0.38	1	4	< 0.001
4. Men with eating disorders have a harder time finding treatment than women.	2.55	0.82	1	4	2.31	0.81	1	4	2.79	0.76	1	4	< 0.001
5. Doctors take eating disorders less seriously in men than in women.	2.66	0.87	1	4	2.37	0.91	1	4	2.93	0.72	1	4	< 0.001
6. There are hardly any specialized treatment services for men with eating disorders.	2.62	0.85	1	4	2.35	0.85	1	4	2.89	0.76	1	4	< 0.001
7. Eating disorders are less treatable for men than for women.	1.93	0.74	1	4	1.85	0.70	1	4	2.00	0.78	1	4	0.023
Total score	2.11	0.43	1	3.86	2.04	0.47	1.00	3.29	2.18	0.38	1.00	3.86	< 0.001

*N* = 242 men. *N* = 249 women. Items were rated on a 4-point scale from 1 (completely disagree) to 4 (completely agree) and aggregated such that higher scores indicated higher stigma-related perceptions of eating disorders in men

Wilcox = Wilcoxon Rank Sum Test for significant difference between genders, all distributions significantly different from normal distribution according to Shapiro-Wilk and Kolmogorov-Smirnov tests

### Data aggregation and analysis

All questionnaires were aggregated per their respective conventions. Variable values are reported as means (*M*) and standard deviations (*SD*) for metric data, and as frequency (*N*) and percent (%) for categorical data.

The reliability of the measures was evaluated using McDonald’s ω [77]. Values above 0.80 indicate good, above 0.70 acceptable, and below 0.50 unacceptable internal consistency [78, 79]. Therefore, although the EDY-Q scores in women showed lower internal consistency, the measure was retained for analysis due to its validation in earlier research.

To assess the bivariate associations between various assessments, we used Pearson correlations. For group comparisons we used t-tests for continuous and chi-square tests for categorical data. Where applicable, effect sizes were estimated. In the case of continuous data, this was Cohen’s *d*, whereby |*d*| ≥ 0.80 reflects a large-sized, 0.79 ≤ |*d*| ≤ 0.21 a medium-sized, and |*d*| ≤ 0.20 a small-sized effect [62]. In the case of categorical data, we computed Cramér’s *V*. A Cramér’s *V* = 0.50 reflects a large-sized effect, *V* = 0.30 a medium-sized effect, and *V* = 0.10 a small-sized effect [62].

Moderation analyses with the various scores for disordered eating symptoms as the independent variable *X*, stigma-related perceptions of EDs in men as the moderator *W*, and help-seeking intentions as the dependent variable *Y* were performed using the PROCESS macro version 4.2 [80], with bootstrapped (5,000 samples) bias-corrected 95% confidence intervals (CI), to ensure the robustness of the estimates and address potential sampling variability. We included sexual orientation as a covariate in the moderation models (coded as orientation towards women, men, women & men, other, not stated), given that members of sexual minority groups show heightened risk for EDs and multiple stigma [81], face unique barriers in help-seeking [82]. Independent and moderator variables were mean-centered. Simple slopes were evaluated at the moderator mean (*M*) and *M* ± 1 *SD* and, more granular, using the Johnson-Neyman technique [83]. We computed moderation analyses separately for men and women focusing on the specific nature of the moderation effect within each gender, instead of facing the complexity of statistically identifying and interpreting three-way interactions [80].

All statistical analyses were performed in IBM SPSS 29 [84]. The threshold for statistical significance in all analyses was defined as *p* < .05. We used case-wise exclusion across analyses for missing data. Plots were created using the ggplot2 package version 3.5.1 [85] in R version 4.4.1 [86] in case of interaction plots for simple slope analysis. To examine the conditional effects of predictor variables at different levels of the moderator, Johnson-Neyman plots were generated using the *johnson_neyman()* function from the interactions package in R [87]. These plots visually depict regions of statistical significance and non-significance across the range of the moderator variable.

## Results

### Sample characteristics

Two individuals were excluded due to implausible weight and age data. Table 2 provides an overview of the sociodemographic characteristics of the samples, along with disordered eating symptoms and intentions to seek help. As expected from the selection of individuals without EDs, the overall level of symptom endorsement was relatively low in both groups. While there were no differences between genders regarding orthorexic and avoidant-restrictive eating symptoms, women endorsed significantly more symptoms measured by the EDE-Q. In contrast, men reported more muscularity-oriented disordered eating symptoms overall and regarding the preoccupation with increasing muscle size (DS) and dissatisfaction regarding their appearance (AI), but not regarding interference with daily functioning (FI). Furthermore, women reported significantly higher levels of stigma-related perceptions of EDs in men and more pronounced help-seeking intentions compared to men. The EDE-Q scores correlated positively with both the scores for muscularity-oriented disordered eating and orthorexic eating in men and women, while no significant correlation was observed with the avoidant-restrictive disordered eating symptom scores. Help-seeking intentions correlated positively with disordered eating symptoms in both genders, except for the avoidant-restrictive disordered eating symptoms. Age in both men and women exhibited a negative correlation with muscle dysmorphic symptoms, but no significant correlation was found with the other eating-related scores (see Table 3). Non-heterosexual women, compared to their heterosexual peers, showed a significantly higher likelihood of seeking help. Apart from that, there were no observable differences in stigma endorsement and the intention to seek help by sociodemographic features within gender groups (see Supplementary Material/Table S2).

**Table 2:** *Sample characteristics*,* disordered eating symptoms*,* stigma-related perceptions*,* and help-seeking intentions*

	Men (*n* = 242)	Women (*n* = 249)	t(df)	X²	*p*	d	V
Parameter	Total	Total					
Age (years)	32.77 (11.07)	28.69 (9.62)	4.36 (476)		< 0.001	0.39	
BMI (kg/m²)	25.12 (3.85)	22.83 (3.77)	6.67 (489)		< 0.001	0.60	
Sexual orientation: Attracted to …							
Women	209 (86.40%)	20 (8.00%)		305.67 (4)	< 0.001		0.79
Men	23 (9.50%)	168 (67.50%)					
Women & men	8 (3.30%)	53 (21.30%)					
Other	1 (0.40%)	0 (0.00%)					
Not stated	1 (0.40%)	8 (3.20%)					
German language proficiency							
First language	216 (89.30%)	207 (83.10%)		3.96 (2)	0.138		0.09
Fluent	23 (9.50%)	36 (14.50%)					
Basic proficiency	3 (1.20%)	6 (2.40%)					
Education							
Less than 12 years	36 (14.90%)	38 (15.30%)		0.01 (1)	0.905		0.01
12 years or more	206 (85.10%)	211 (84.70%)					
Migration background							
Yes	54 (22.30%)	72 (28.90%)		2.80 (1)	0.094		0.08
No	188 (77.70%)	177 (71.10%)					
Marital status							
Single	170 (70.20%)	194 (77.90%)		4.00 (2)	0.136		0.09
Married	66 (27.30%)	49 (19.70%)					
Divorced	6 (2.50%)	6 (2.40%)					
Widowed	0 (0.00%)	0 (0.00%)					
Living circumstances							
One-person household	67 (27.70%)	66 (26.50%)		1.05 (2)	0.593		0.05
Multi-person household	175 (72.30%)	182 (73.10%)					
Not stated	0 (0.00%)	1 (0.40%)					
EDE-Q	1.28 (1.02)	1.72 (1.31)	− 4.16 (467)		< 0.001	-0.37	
DOS	18.04 (5.14)	18.56 (5.24)	− 1.11 (489)		0.267	− 0.10	
EDY-Q^a^	1.29 (0.81)	1.25 (0.73)	0.69 (478)		0.488	0.06	
EDY-Q FAED^a^	1.38 (1.10)	1.42 (0.99)	− 0.45 (463)		0.657	− 0.04	
EDY-Q SE^a^	1.91 (1.42)	1.90 (1.52)	0.04 (478)		0.972	0.00	
EDY-Q FD^a^	0.24 (0.79)	0.31 (0.77)	− 0.91 (478)		0.365	− 0.08	
MDDI	27.75 (8.38)	25.41 (7.05)	3.34 (470)		0.001	0.30	
MDDI DS	12.05 (4.62)	8.98 (3.73)	8.10 (463)		< 0.001	0.73	
MDDI AI	8.60 (3.68)	9.84 (3.89)	− 3.61 (489)		< 0.001	− 0.33	
MDDI FI	7.1 (3.27)	6.60 (3.04)	1.76 (489)		0.079	0.16	
Stigma	2.04 (0.47)	2.18 (0.38)	− 3.64 (460)		< 0.001	− 0.33	
Help-Seeking Intentions (SOCQ-ED)	0.38 (0.98)	0.90 (1.77)	− 4.03 (390)		< 0.001	− 0.36	

Values are shown in *n* (%) or means and standard deviations (in brackets). *Min* Minimum Value, *Max* Maximum Value, *EDE-Q* Eating Disorder Examination-Questionnaire, *DOS* Duesseldorf Orthorexia Scale, *EDY-Q* Eating Disorders in Youth-Questionnaire, *FAED* food avoidance emotional disorder, *SE* selective eating, *FD* functional dysphagia, *MDDI* Muscle Dysmorphic Disorder Inventory, *DS* drive for size, *AI* appearance intolerance, *FI* functional impairment, *Stigma* Stigma-related perceptions of eating disorders in men, *SOCQ-ED* Stages of Change Questionnaire for Eating Disorders

^a^*N* = 232 in men and *N* = 248 in women due to missing responses

**Table 3:** *Bivariate variable correlations for the men and women sample*

Variable	1	2	3	4	5	6	7	8
1. Age	1	0.24**	0.03	− 0.04	− 0.04	− 0.14*	− 0.03	− 0.03
2. BMI	0.32**	1	0.50**	0.05	− 0.23**	0.07	− 0.02	0.07
3. EDE-Q	0.08	0.44**	1	0.52**	0.10	0.56**	0.12	0.20**
4. DOS	− 0.07	0.04	0.50**	1	0.13*	0.52**	0.03	0.19**
5. EDY-Q^a^	− 0.12	− 0.26**	0.06	0.15*	1	0.28**	0.04	0.00
6. MDDI	− 0.17**	− 0.02	0.58**	0.49**	0.39**	1	0.06	0.24**
7. Stigma	− 0.11	− 0.14*	0.08	0.21**	0.09	0.20**	1	0.01
8. SOCQ-ED	0.04	0.16*	0.36**	0.26**	0.04	0.16*	− 0.03	1

Men sample presented below and women sample above the diagonal, *N*_men_ 242, *N*_women_ 249, *BMI* Body Mass Index, *EDE-Q* Eating Disorder Examination-Questionnaire, *DOS* Duesseldorf Orthorexia Scale, *EDY-Q* Eating Disorders in Youth-Questionnaire, *MDDI* Muscle Dysmorphic Disorder Inventory, *Stigma* Stigma-related perceptions of eating disorders in men

Pearson correlations with pairwise case exclusion applied

^a^*N* = 232 for men sample and *N* = 248 for women sample due to missing responses

*p < .05. ** *p* < .01

### Men’s ED-stigma and help-seeking in men

**Fig. 1:**
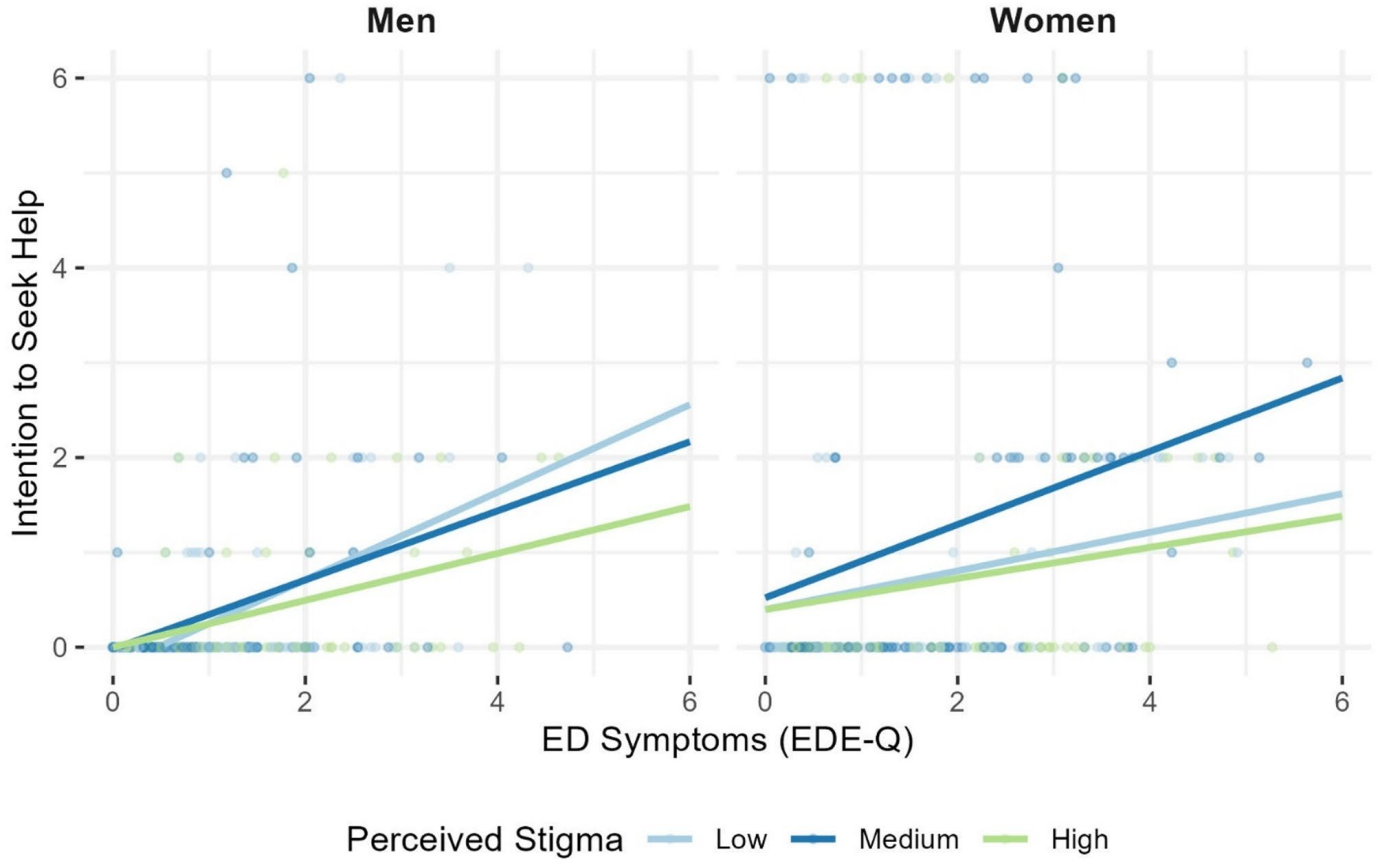
*Moderating effect of stigma-related perceptions of eating disorders in men on the* association *between eating disorder symptom severity and help-seeking intentions by gender.* The figure illustrates the linear association between eating disorder (ED) symptoms and the intention to seek help for an ED, moderated by stigma-related perceptions of EDs in men among both men and women. The lines represent low, medium, and high levels of perceived stigma, categorized into tertiles for visualization purposes. *ED* eating disorder, *EDE-Q* Eating Disorder Examination-Questionnaire

**Fig. 2:**
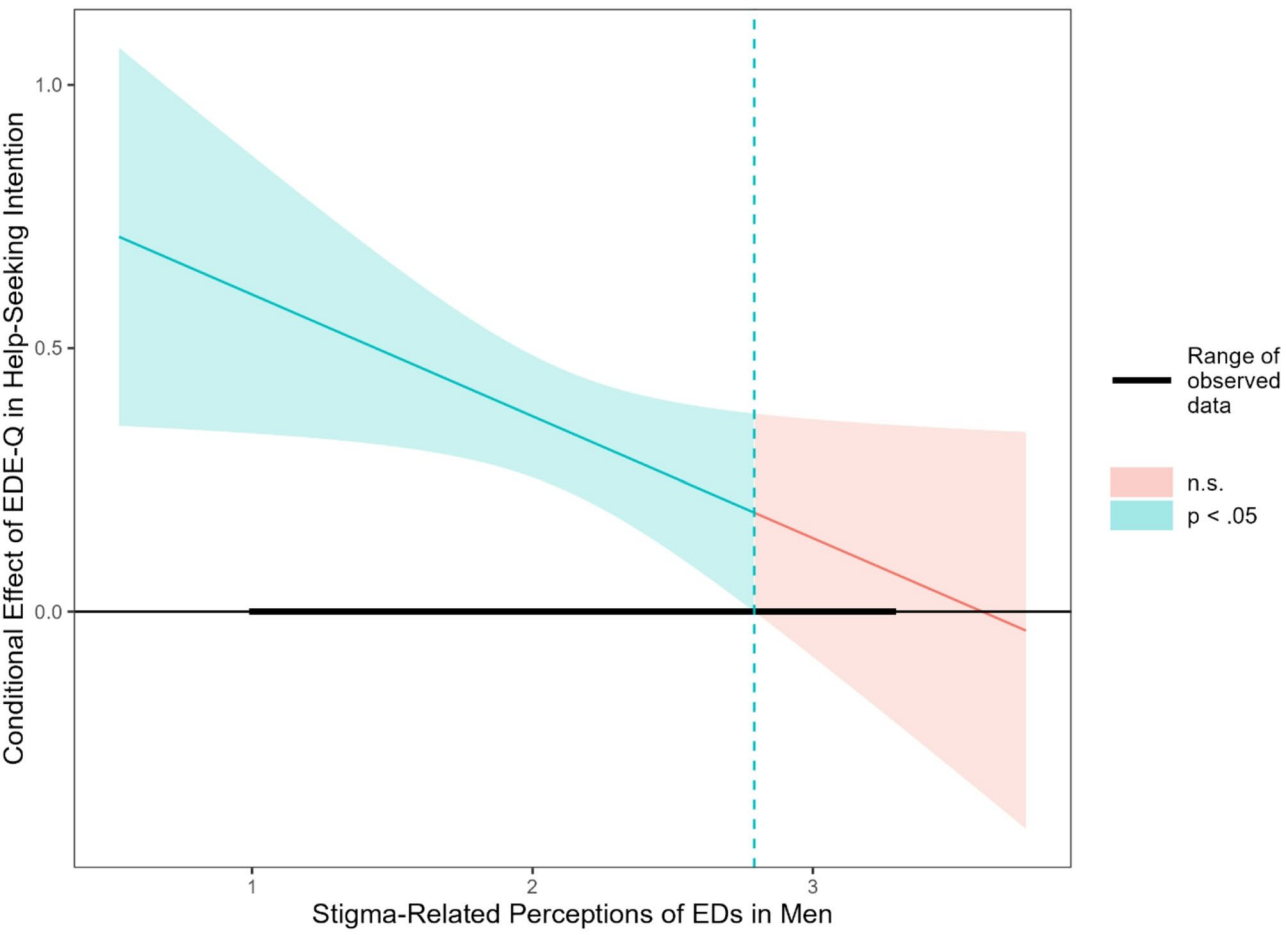
*Johnson-Neyman significance regions for the conditional effect of symptom severity on help-seeking intentions at different values of stigma-related perceptions of eating disorders in men for the men sample.* The effect is significant at stigma-related perceptions values below the dashed, vertical line (2.79 on a scale of 1 to 4). *ED* eating disorder, *EDE-Q* Eating Disorder Examination-Questionnaire

The moderator analysis with EDE-Q scores as the independent variable, stigma-related perceptions of EDs in men as the moderator, and help-seeking intentions as the dependent variable, while controlling for participants’ sexual orientation as a covariate, yielded significant results, *R*² = 0.15. As hypothesized, higher EDE-Q scores correlated positively with enhanced help-seeking intentions. Stigma-related perceptions were not associated with help-seeking intentions. However, and as expected, stigma-related perceptions moderated the association of symptom severity and help-seeking intentions in men, *R*²_change_ = 0.02. Consistent with our hypotheses, simple slope analysis (see Fig. 1) showed that the association between symptom severity and help-seeking intentions diminished as levels of stigma-related perceptions increased, *b*_− 1SD_ = 0.47, *b*_M_ = 0.36, *b*_+ 1SD_ = 0.25. Although this moderation effect remained significant throughout the evaluated mean ± 1 *SD* levels, all *p*s ≤ 0.001, the more detailed Johnson-Neyman analysis indicated that EDE-Q scores were significantly associated with help-seeking intentions only up to a stigma-related perceptions score of 2.79, beyond which the association became non-significant (see Fig. 2).

Additionally, we investigated the moderating effects of stigma-related perceptions on the associations between other disordered eating symptoms and help-seeking intentions (see Table 4). Regarding orthorexic symptoms, the independent variable, moderator, and their interaction accounted for significant variance in help-seeking intentions, *R*² = 0.08. Higher orthorexic symptom scores correlated with increased help-seeking intentions. However, stigma-related perceptions were neither associated to help-seeking intentions, nor did they moderate the impact of orthorexic symptoms on help-seeking intentions. The model employing muscle dysmorphic disordered eating scores as the independent variable also accounted for significant variance in help-seeking intentions, *R*² = 0.04. Higher muscle dysmorphic symptoms scores were positively associated with help-seeking intentions. Stigma-related perceptions independently were not associated with help-seeking intentions, and did not moderate the effect of the muscle dysmorphic symptoms on the intention to seek help. Finally, avoidant/restrictive disordered eating symptoms together with stigma and their interaction did not account for significant variance, *R*² = 0.04. These symptoms, thus, were unrelated to help-seeking intentions and were not moderated by stigma-related perceptions. In summary, we identified a moderating effect of stigma-related perceptions specifically on the association between intentions to seek help and ED symptoms measured by the EDE-Q in our men´s sample. This effect was not observed for symptoms assessed by any other measurement instrument in this group.

**Table 4:** *Results of moderation analyses for Stigma-Related perceptions*

	Men (*n* = 242)							Women (*n* = 249)						
	Model estimates			Parameter estimates				Model estimates			Parameter estimates			
	*F*	*df1*,* df2*	*p*	*b*	*SE*	*p*	95% CI [*LL*,* UL*]	*F*	*df1*,* df2*	*p*	*b*	*SE*	*p*	95% CI [*LL*,* UL*]
EDE-Q	10.17	4, 237	< 0.001**					2.77	4, 244	0.028*				
EDE-Q				0.36	0.08	< 0.001**	0.21, 0.50				0.26	0.07	0.003*	0.12, 0.40
Stigma				− 0.03	0.16	0.808	− 0.33, 0.29				− 0.07	0.26	0.813	− 0.59, 0.45
EDE-Q x Stigma				− 0.23	0.15	0.037*	− 0.50, − 0.09				0.02	0.22	0.918	− 0.32, 0.53
Covariate				0.000	0.17	0.997	− 0.29, 0.41				0.10	0.11	0.343	− 0.92, 0.33
DOS	4.90	4, 237	0.001*					2.57	4, 244	0.039*				
DOS				0.06	0.02	< 0.001**	0.02, 0.09				0.06	0.02	0.003*	0.02, 0.11
Stigma				− 0.16	0.18	0.235	− 0.51, 0.19				0.00	0.31	1.000	− 0.60, 0.60
DOS x Stigma				− 0.03	0.04	0.269	− 0.09, 0.05				0.01	0.06	0.863	− 0.10, 0.12
Covariate				− 0.04	0.18	0.800	− 0.34, 0.39				0.12	0.11	0.273	− 0.10, 0.33
EDY-Q^a^	2.27	4, 227	0.063					0.36	4, 243	0.837				
EDY-Q				0.05	0.09	0.513	− 0.13, 0.22				− 0.02	0.13	0.916	− 0.27, 0.24
Stigma				− 0.11	0.18	0.441	− 0.48, 0.22				0.04	0.26	0.889	− 0.49, 0.53
EDY-Q x Stigma				0.42	0.27	0.006	− 0.10, 0.89				0.06	0.31	0.895	− 0.53, 0.68
Covariate				− 0.14	0.21	0.349	− 0.52, 0.31				0.13	0.11	0.237	− 0.07, 0.37
MDDI	2.38	4, 237	0.053					3.94	4, 244	0.004*				
MDDI				0.02	0.01	0.011*	0.01, 0.03				0.06	0.02	< 0.001**	0.03, 0.09
Stigma				− 0.18	0.18	0.206	− 0.55, 0.17				− 0.03	0.25	0.928	− 0.54, 0.45
MDDI x Stigma				0.02	0.02	0.150	− 0.01, 0.05				0.02	0.03	0.732	− 0.05, 0.08
Covariate				− 0.06	0.20	0.679	− 0.40, 0.37				0.10	0.11	0.354	− 0.09, 0.33

Values were computed with PROCESS version 4.2 model 1 using bootstrapping procedure with 5,000 iterations and mean centering of predictor and moderator variables. Respective symptom severity was included as independent variable, intention to seek help as dependent variable; *Stigma* mean score of stigma-related perceptions of eating disorders in men items as moderator; *Covariate* sexual orientation. *EDE-Q* Eating Disorder Examination-Questionnaire; *DOS* Duesseldorf Orthorexia Scale; *EDY-Q* Eating Disorders in Youth-Questionnaire; *MDDI* Muscle Dysmorphic Disorder Inventory; *CI* confidence interval; *LL* lower limit; *UL* upper limit

^a^*N* = 232 for men and *N* = 248 for women due to missing responses

*p < .05. **p < .01

### Men’s ED-stigma and help-seeking in women

In the women’s sample, the model, which included ED symptoms assessed by the EDE-Q as independent variable, also accounted for significant variance in help-seeking intentions, *R*² = 0.04. As assumed, higher EDE-Q scores were positively correlated with an increase in help-seeking intentions, and stigma-related perceptions were not associated with help-seeking intentions. In contrast to the men´s sample, but as expected and consistent with the gender-specificity hypothesis, stigma-related perceptions did not moderate the association between symptom severity and help-seeking intentions among women, *R*²_change_ = 0.00 (Fig. 1).

We again examined whether stigma-related perceptions moderated the associations of the other disordered eating symptoms with help-seeking intentions within the women’s sample. (see Table 4). The model incorporating orthorexic symptoms as independent variable accounted for significant variance in help-seeking intentions, *R*² = 0.04. Specifically, higher orthorexic symptoms scores were associated with an increase in help-seeking intentions. Conversely, stigma-related perceptions were neither themselves associated with help-seeking intentions, nor did they moderate the association between orthorexic symptoms and help-seeking. The model including muscle dysmorphic disordered eating symptoms as independent variable also accounted for significant variance of intentions to seek help, *R*² = 0.06. Data revealed that elevated muscle dysmorphic disordered eating symptoms scores were associated with increased intentions to seek help. However, neither were stigma-related perceptions associated with greater help-seeking intentions, nor did they moderate the association between muscle dysmorphic disordered eating symptoms and help-seeking intentions. Finally, the avoidant/restrictive disordered eating symptoms scores exhibited no significant association with help-seeking intentions and were not influenced by stigma-related perceptions in the women´s sample. Consequently, we found no evidence to suggest that stigma-related perceptions of EDs in men moderated the association of disordered eating symptoms, as measured by any instrument, on help-seeking intentions within the women´s sample.

## Discussion

EDs are severe psychiatric conditions affecting individuals across all genders [11], though men remain underrepresented in specialized treatment settings [16] due to stigma and lower help-seeking rates [17, 38]. This study explored whether there is a gender-specific facet of ED stigma, which uniquely impacts men’s intentions to seek help for disordered eating symptoms, focusing on differences in symptom presentation and stigma’s potential role in reducing help-seeking across genders. We therefore examined whether stigma-related perceptions of EDs in men moderate the association between symptom severity and help-seeking intentions, assuming that the proposed stigma facet moderates this association specifically in men but not in women.

We found that both men and women exhibited greater intentions to seek help as the severity of perceived ED symptoms increased across a wide range of symptoms. The inclusion of these symptoms was guided by prior findings indicating that disordered eating in men is associated with muscularity-oriented, orthorexic, and avoidant/restrictive behaviors [22, 88, 89]. The treatment motivating impact of perceived ED symptoms was significant in all models except for avoidant/restrictive eating behavior in both genders, and muscularity-oriented disordered eating in men. The former could be related to the low reliability of the EDY-Q found in this and other studies [52, 72]. The latter could be interpreted as supporting the notion of a “feminized” social construction of EDs and its assumed contribution to stigma. While women are exposed to greater social pressures towards thinness, men are increasingly subject to cultural pressures to achieve a lean yet muscular body, a body image ideal also known as the mesomorphic body ideal [90], which contribute*s* to body image concerns, disordered eating, and symptoms characteristic of both EDs and muscle dysmorphia [91]. This mesomorphic ideal is not captured by traditional ED measures and differs from the dominating women-centered construction of EDs. Muscularity-oriented ED symptoms may thus result in less distress and, consequently, lower help-seeking intentions in men, as they align with the socially desirable mesomorphic (“male”) body ideal, not typically associated with EDs in the public’s perception [92, 93]. Consistent with previous findings [52] and our hypotheses, the impact of ED symptoms on help-seeking intentions decreased as stigma-related perceptions of EDs in men increased, but only in men and specifically for “feminized” ED symptoms such as thinness and weight concerns (assessed via the EDE-Q). By contrast, no moderating effect of stigma was observed in regarding other symptom domains or among women, highlighting the gender-specific nature of this stigma facet in a double manner. First, the exclusive occurrence with “feminized” (i.e., thinness-oriented) ED symptoms could be related to the fact that such symptoms in men violate masculine gender roles. EDs are commonly perceived, i.e., socially constructed, as “women’s disorders”. This manifests in newspapers, movies, and social media content [94, 95], and can also be observed in affected men themselves [96] and their related others [97, 98]. Second, the finding that only men are affected indicates the gender-specificity of the assessed stigma construct. The finding that women, compared to men, reported higher levels of perceived ED stigma towards men aligns with a body of literature that–despite some heterogeneity–has similarly observed that women in various countries and contexts show increased sensitivity to stigma [99–101]. As Kulesza et al. [99] discuss, this may be a result of women’s greater exposure to gender-based discrimination in everyday life, which could sensitize them to other forms of stigmatization such as mental health stigma. Alternatively, this finding may be linked to sex differences in the adherence to gender norms. An individual’s conformity to masculine norms – which is commonly higher in men – was found to predict higher ED stigmatization towards others [49]. Accordingly, men may be less likely to endorse stigma facets that conflict with male gender norms or due to social desirability. The gender-specific effect of the assessed men’s ED stigma construct on men’s help-seeking found in our study is in line with gender theories [47, 50, 51], and supports the findings from previous research highlighting that men with EDs may face unique challenges in the help-seeking process, with stigma as a particular barrier [38, 39].

These findings have implications across multiple domains. On a governmental level, they can inform a human-rights based approach to healthcare [102] due to the identification of a treatment barrier for men that needs to be reduced. Further on, the results point towards the extension of existing interventions to reduce stigma in EDs that do not adequately address the perspective of men [103]. There is evidence that medical staff and researchers are not immune to the misconception of EDs as a “women’s disorder”. Men with EDs repeatedly report facing minimization and ignorance of their ED symptoms by medical staff [76]. Thus, our results add to the claim for the enrichment of specialized individual and community level treatment and self-help programs with men’s unique perspectives (e.g., self-stigmatization, masculinity ideals). Finally, on a theoretical level, our findings support a complex and multidimensional understanding of stigma and its potential intersection with aspects of gender.

## Strengths and limitations

While the data supports several of our assumptions and aligns with previous research, it is essential to acknowledge some limitations. First, we assessed a general population sample and excluded individuals who had ever received a formal ED diagnosis or significant higher-weight status/obesity. This decision was made to specifically examine the effects of a gender-related facet of stigma associated with EDs in men, without potential confounding influences from prior ED-related treatment experiences or other dimensions of stigma, such as weight-related stigma. Thus, the present findings need to be replicated in a sample of individuals with EDs to demonstrate comprehensive validity. Second, we opted for a specific conceptual focus on stigma and help-seeking, whereby both are complex, multidimensional concepts. While the study’s specific conceptual focus and the economic method of assessment can be considered strengths, the use of single-item measures and a narrowly defined scope may increase the risk of distortion and limit generalizability. Future studies should therefore endeavor to develop and implement more comprehensive psychometric measures that can capture stigma and help-seeking in their multidimensionality and systematically target underlying processes. To date, there is a lack of such validated instruments for use in German-speaking countries. Our set of stigma items represents an effort in this direction, as it directly reflects the lived experiences of men with EDs [76], an underrepresented and under-researched group in EDs [104]. Third, we opted to include the EDY-Q and the stigma measure despite their lower internal consistency in our women’s sample, given their prior validation and established relevance for assessing the targeted constructs in previous research. Nonetheless, the limited internal consistency should be considered when interpreting the findings. Fourth, we examined the impact of stigma using a cross-sectional design and regarding the intent to seek but not the actual seeking of help, which restricts the ability to draw causal inferences from the observed associations especially regarding the actual seeking of help. Future research should continue to assess these relations longitudinally to assess whether help was sought, and what factors may hinder such decisions. Additionally, this study is based on a secondary analysis of data compiled from three distinct online survey datasets. Despite careful efforts to ensure consistency and accuracy, the possibility of data distortion cannot be fully ruled out. As such, future research should replicate these analyses using an independent primary data collection, alongside comprehensive data quality assurance procedures. Fifth, we conducted separate moderation analyses for men and women to examine the specific nature of the moderation effects within each gender, avoiding the complexity associated with statistically identifying and interpreting three-way interactions [80]. To gain a more nuanced understanding of how interactions between moderators may differ across genders (i.e., moderated moderation), future studies should aim to recruit larger samples, enabling more robust and detailed analyses, and assess a broader range of potential sociodemographic covariates. Sixth, although our approach accounts for specific diversity factors to foster clearer interpretation, it omits others (e.g., gender-queerness, race/ethnicity), limiting conclusions regarding intersectionality. Future research should explore additional eating disorder symptoms (e.g., binge eating) and examine diversity dimensions in greater depth, with particular attention to their complex intersectional interrelations.

## Conclusions

EDs are an often underrecognized but increasingly significant health concern in men. Our findings highlight perceived stigma of EDs in men as a potential help-seeking barrier for men, discouraging help-seeking intentions and possibly impacting access to timely and specialized treatment. This applies to “traditional” or “feminized” ED symptoms, which may conflict with masculine gender norms. Addressing such stigma of EDs in men is crucial for improving health outcomes and promoting gender equity in mental health care. Future interventions and health campaigns should focus on further dismantling and reducing ED stigma to ensure equitable access to ED diagnosis, treatment, and support for all individuals.

## Supplementary Information

Below is the link to the electronic supplementary material.

Supplementary Material 1.

## Data Availability

The materials and data that support the findings of this study are available from the corresponding author upon reasonable request.

## Abbreviations

AI: Appearance intolerance
ARFID: Avoidant/restrictivefood intake disorder
DOS: Duesseldorf orthorexia scale
DS: Drive for size
ED: Eating disorder
EDE-Q: Eating disorder examination-questionnaire
EDY-Q: Eating disorders in youth-questionnaire
FI: Functional impairment
M: Mean
MDDI: Muscle dysmorphic disorder inventory
SD: Standard deviation
SOCQ-ED: Stages of change questionnaire for eating disorders
