# Supplementary material for: Boys don’t try? Gendered stigma specifically reduces help-seeking for disordered eating in men, but not women
Source: J Eat Disord. 2025 Sep 17;13:204. doi: 10.1186/s40337-025-01407-7 (PMC12442273; doi:10.1186/s40337-025-01407-7)
Supplement: Supplementary file 1 — Supplementary Material 1. [file 40337_2025_1407_MOESM1_ESM.docx]

**Supplementary Material**

**Table S1**

*Reliability Measures (McDonald’s Omega) of all Questionnaires and Subscales*

| **Instrument** | **Men (*n* = 242)** | **Women (*n* = 249)** |
| --- | --- | --- |
| EDE-Q | .93 | .96 |
| DOS | .85 | .86 |
| EDY-Q^a^ | .66 | .55 |
| EDY-Q FAED^a^ | .52 | .41 |
| EDY-Q SE^a^ | .68 | .76 |
| EDY-Q FD^a^ | .92 | .84 |
| MDDI | .82 | .70 |
| MDDI DS | .88 | .79 |
| MDDI AI | .83 | .84 |
| MDDI FI | .74 | .73 |
| Stigma | .74 | .64 |

*Note*. EDE-Q = Eating Disorder Examination-Questionnaire; DOS = Duesseldorf Orthorexia Scale; EDY-Q = Eating Disorders in Youth-Questionnaire; FAED = food avoidance emotional disorder; SE = selective eating; FD = functional dysphagia; MDDI = Muscle Dysmorphic Disorder Inventory; DS = drive for size; AI = appearance intolerance; FI = functional impairment; Stigma = Stigma-related perceptions of eating disorders in men; Stigma 4 to 7 = mean score of Stigma based on items 4 to 7.

^a^ *N* = 232 in men and *N* = 248 in women due to missing responses.

**Table S2**

*Perceived Stigma and Help-Seeking Intentions by Sociodemographic Characteristics*

|  | **Men (*n* = 242)** | | | | |  | **Women (*n* = 249)** | | | | |
| --- | --- | --- | --- | --- | --- | --- | --- | --- | --- | --- | --- |
| **Educational Attainment** | **< 12 years  (*n* = 36)** | **> 12 years  (*n* = 206)** | ***t (df)*** | ***p*** | ***d*** |  | **< 12 years  (*n* = 38)** | **> 12 years  (*n* = 211)** | ***t (df)*** | ***p*** | ***d*** |
| Stigma | 2.08 (0.56) | 2.03 (0.46) | 0.51 (240) | .613 | 0.09 |  | 2.07 (0.38) | 2.20 (0.37) | -1.93 (247) | .055 | -0.34 |
| SOCQ-ED | 0.50 (1.06) | 0.35 (0.97) | 0.82 (240) | .413 | 0.15 |  | 0.95 (1.74) | 0.89 (1.78) | 0.20 (247) | .845 | 0.03 |
| **Marital Status** | **single/divorced  (*n* = 176)** | **married  (*n* = 66)** | ***t (df)*** | ***p*** | ***d*** |  | **single/divorced  (*n* = 200)** | **married  (*n* = 49)** | ***t (df)*** | ***p*** | ***d*** |
| Stigma | 2.06 (0.49) | 1.97 (0.43) | 1.29 (240) | .197 | 0.19 |  | 2.20 (0.37) | 2.09 (0.38) | 1.92 (247) | .056 | 0.31 |
| SOCQ-ED | 0.36 (0.94) | 0.42 (1.10) | -0.47 (240) | .641 | -0.07 |  | 0.88 (1.79) | 0.96 (1.72) | -0.28 (247) | .780 | -0.05 |
| **Living Situation** | **alone  (*n* = 67)** | **with others  (*n* = 175)** | ***t (df)*** | ***p*** | ***d*** |  | **alone  (*n* = 66)** | **with others  (*n* = 182)** | ***t (df)*** | ***p*** | ***d*** |
| Stigma | 2.02 (0.52) | 2.04 (0.46) | -0.30 (240) | .763 | -0.04 |  | 2.17 (0.40) | 2.18 (0.37) | -0.09 (246) | .926 | -0.01 |
| SOCQ-ED | 0.24 (0.70) | 0.43 (1.07) | -1.62 (182.01) | .108 | -0.19 |  | 0.76 (1.59) | 0.95 (1.84) | -0.76 (246) | .450 | -0.11 |
| **Sexual Orientation** | **non-heterosexual  (*n* = 32)** | **heterosexual  (*n* = 209)** | ***t (df)*** | ***p*** | ***d*** |  | **non-heterosexual  (*n* = 73)** | **heterosexual  (*n* = 168)** | ***t (df)*** | ***p*** | ***d*** |
| Stigma | 1.14 (0.48) | 2.02 (0.47) | -1.29 (239) | .200 | -0.24 |  | 2.23 (0.33) | 2.16 (0.39) | -1.24 (239) | .218 | -0.17 |
| SOCQ-ED | 0.63 (1.34) | 0.34 (0.92) | -1.17 (35.59) | .252 | -0.29 |  | 1.30 (2.01) | 0.75 (1.67) | -2.05 (117.03) | .042 | -0.31 |

*Note.* Values show means and standard deviations (in parentheses). Stigma = Stigma-related perceptions of eating disorders in men; SOCQ-ED = Stages of Change Questionnaire for Eating Disorders.
